# The Inverted Circuit: Attribution, Answerability, and Accountability in Canadian Health Governance

**DOI:** 10.1111/1468-0009.70126

**Published:** 2026-09-11

**Authors:** ALAN H. MENKIS

**Affiliations:** ^1^ University of Manitoba; ^2^ formerly Winnipeg Regional Health Authority and St. Boniface Hospital and Health Sciences Centre

**Keywords:** health governance, accountability, fiduciary duty, attribution, answerability, patient safety, outcome measurement, health policy

## Abstract

**Policy Points**

- Canada and the United States have not failed for want of accountability mechanisms in health governance. They have failed because accountability is repeatedly invoked where attribution and answerability are not already standing, so the result is the punishment of visible individuals rather than structural correction.
- The corrective is to restore the order of dependency: first name which institution is responsible for an outcome, then require it to report against a benchmarked standard set in advance, and only then attach consequences. Applied in this order, the framework transfers to any federated system in which central funding flows without binding accountability for results.
- Structural correctives require independent oversight bodies whose mandate, funding, and membership are insulated from the institutions they regulate; proactive mandatory reporting against pre‐set standards; and explicit attribution of population‐level outcomes to named institutional responsibilities.

**Context:**

Canada and the United States have pursued health‐system accountability for decades through inquiries, commissions, and public reporting, yet the same governance failures recur. This Perspective argues that accountability has repeatedly been reached for before the conditions that make it coherent were in place.

**Methods:**

The paper develops a conceptual framework, the fiduciary circuit. The analysis draws on inquest and commission records, the safety‐science and governance literature, and illustrative program‐ and system‐level cases from cardiac care and intergovernmental health financing in Canada and the United States.

**Findings:**

Attribution, answerability, and accountability stand in a relation of logical dependency rather than temporal sequence: accountability is incoherent unless responsibility has first been assigned and answerability secured. Where the circuit is closed, as in Manitoba's CODE STEMI program for acute heart‐attack care, outcomes improve; where it is entered at accountability after a failure, as in federal‐provincial transfers, it produces the scapegoating of visible individuals while structural conditions persist. The recurring signature is that improvement which is not structurally protected is not retained.

**Conclusions:**

The corrective is not another comprehensive reform but Popperian piecemeal engineering paired with independent statutory bodies whose mandate, funding, and membership are insulated from the institutions they regulate. Naming who is responsible, requiring them to answer against standards set in advance, and only then holding them to account is the order the fiduciary tradition has always demanded.

On september 19, 2008, brian sinclair wheeled himself into the emergency room at the Winnipeg Health Sciences Centre. A clinic had referred him there with a note explaining his condition: a blocked catheter. He spoke briefly with a triage aide. He waited. Thirty‐four hours later he was found dead in his wheelchair. He had died of a treatable bladder infection. He was never formally entered into the system. No one had asked him if he was waiting to be seen.

The inquest that followed, presided over by Provincial Court Judge Timothy Preston, ran for 40 hearing days, heard from 82 witnesses, and produced 63 recommendations. Judge Preston concluded plainly: he did not have to die.

Thirteen years earlier, at the same institution, Associate Chief Judge Murray Sinclair, no relation to Brian Sinclair, presided over an inquest into the deaths of 12 children in the Winnipeg pediatric cardiac surgery program. His report, released in 2000, identified the same structural gaps in governance and accountability. Between those two inquests, an independent expert review, the Koshal Report of 2003, documented the same gaps again. A University of Ottawa Heart Institute consultation in 2014 confirmed them a third time. Two judicial inquiries. Three independent reviews across two decades. The same institution. The same governance gaps. The same finding: this did not have to happen. The same recommendations. The same nonimplementation at the structural level.

## 1 The Diagnostic: A Problem of Sequence

This pattern is not unique to Manitoba, and it is not unique to Canada. The Institute of Medicine's landmark report, *To Err I.s Human* (1999), estimated that as many as 98,000 Americans died annually from preventable medical errors.^1^ The report was celebrated, commissioned responses were constituted, and the structural governance architecture that would have made its accountability recommendations enforceable was not built. The *Crossing the Quality Chasm* report followed in 2001 with the same finding restated.^2^ A subsequent analysis published in 2013 estimated the annual toll at between 210,000 and 400,000 preventable deaths, making medical errors the third leading cause of death in the United States, a finding that prompted congressional testimony but no structural governance corrective.^3^ A generation later, the Centers for Medicare and Medicaid Services quality reporting infrastructure measures process indicators with sophistication while binding outcome accountability at the federal level remains structurally absent. The pattern persists: accountability demanded without prior attribution, answerability performative rather than structural, the conditions generating preventable harm undisturbed.

These efforts were not failures. They produced real and consequential gains in safety culture and in measured process. What they did not produce, in either country, was the structural corrective: an independent body empowered to attribute population‐level outcomes and to enforce consequences for nonperformance. Improvement that is not structurally protected is not retained, and so the gains were absorbed and the same failures recurred. The Institute for Healthcare Improvement campaigns scaled from the 100,000 Lives Campaign to the 5 Million Lives Campaign;^4^ while the later statistical tracking was widely regarded as inconclusive, the patient‐safety culture they established endured. The gains were real and some endured. What was never installed was the structural corrective, and so the system‐level failures recurred.

Canada's health care system has not failed for want of accountability mechanisms. It has failed because those mechanisms have been reached for before the conditions that make them coherent were in place. This paper proposes the fiduciary circuit as the corrective framework. The term is used here in its original and broader sense: the duty owed by anyone who holds power in trust for another. In its clinical form, that duty has two parts: the duty of competence and the duty to place the patient's interest first; the financial trustee is the most familiar instance, not the whole of the meaning. The paper argues for the structural conditions under which the circuit can be made operational and identifies the institutional architecture of resistance that has prevented its implementation across three decades of reform.

## 2 The Fiduciary Circuit: Three Functions in a Relation of Dependency

**Figure 1:**
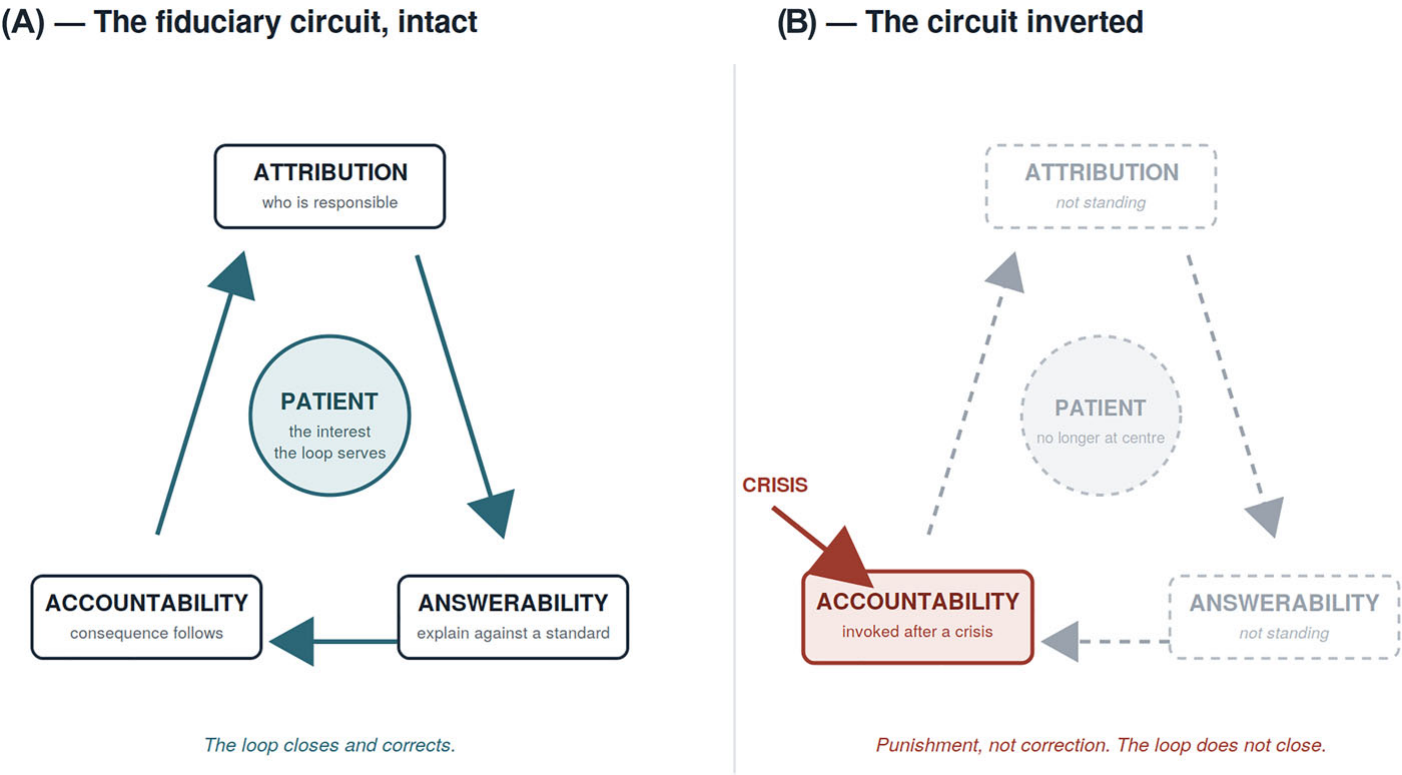
The Fiduciary Circuit and Its Inversion. (A) When attribution and answerability are standing, the three functions form a continuous loop that serves the patient and corrects itself. (B) When the loop is entered at accountability after a crisis, without standing attribution or answerability, it does not close, and the result is the scapegoating of the most visible individual rather than structural correction. Figure rendered with the assistance of Claude Opus 4.8 (Anthropic), accessed June 2026, from the author's design and specification. All conceptual content, structure, and clinical accuracy were determined and verified by the author. [Colour figure can be viewed at wileyonlinelibrary.com]

The framework proposed here rests on three functions: attribution, answerability, and accountability. They are not synonyms, and their conflation is a consequential and largely underexamined confusion in health governance. The claim is not that the three are performed in temporal order, one after another in time. It is a claim of logical dependency: accountability is coherent only when attribution and answerability are already standing. To hold an institution to account for an outcome it was never assigned, and was never required to explain, is not accountability out of order; it is incoherent, and in practice it becomes the scapegoating of the most visible individual after a crisis. The three are best understood as overarching characteristics of a governance arrangement that stand in a relation of dependency. The circuit is the continuous loop these functions form when all three are present. It is inverted when it is entered at the accountability node after a failure, rather than grounded in standing attribution and answerability (Figure 1).

Attribution is the assignment of responsibility for decisions and outcomes to the institutions and offices positioned to affect them. Attribution is not blame. It is the prerequisite for clarity. Reason^5^ distinguished the sharp end, the clinician who makes the immediate decision, from the blunt end, the administrator, the government, or the governance architecture that shaped the conditions within which that decision was made. A system that attributes only at the sharp end while leaving the blunt end unexamined has not completed the fiduciary circuit. It has merely located the most visible person in the chain. Attribution, when properly practiced, runs to both ends.

In Canadian health care governance, attribution is structurally discouraged by the federated design. The diffusion of responsibility across federal transfers, provincial administration, and institutional delivery means that no level is assigned the population outcome; the federal–provincial transfer agreements set targets without assigning consequence. The complexity of that architecture, combined with the institutional interests of hospitals, professional bodies, academia, and governments, creates conditions in which responsibility is perpetually diffuse. Governments blame providers; providers blame governments; institutions blame underfunding; everyone agrees the system is the problem. The system, of course, is an abstraction. It cannot be held to account. This is not an accident. It is the institutional expression of a governance architecture that, consciously or not, protects incumbents from the scrutiny that genuine attribution would impose. The parallel to US federal health governance is close: when the Centers for Medicare and Medicaid Services transfers funds to states without binding outcome accountability, and when states in turn transfer resources to health systems without structural attribution requirements, the same diffusion of responsibility is produced by the same structural design.

The attribution gap in the United States is well documented. A 2016 report commissioned by the Department of Health and Human Services examined attribution as a fundamental challenge in value‐based purchasing, noting that “numerous clinicians and facilities are often involved in a patient's care making it difficult to know who is responsible for the patient's outcomes.”^6^ The report provided guiding principles and a selection framework for attribution models. The structural condition it described has not changed. Attribution remains, in US federal health governance as in Canadian provincial governance, a documented and unresolved problem, not for want of analysis but for want of the institutional architecture required to make it consequential.

Answerability is the standing obligation of those who hold authority in trust to explain their decisions and outcomes, on a defined schedule and against standards set in advance and benchmarked to accepted national and international norms, to those on whose behalf the authority is exercised. It is not transparency as a communications strategy, and it is sharply distinct from reactive disclosure produced only when a crisis demands it. It is the genuine willingness to submit reasoning to scrutiny, to be questioned, and to revise in light of what the questioning reveals. Ingelfinger^7^ wrote in 1980 about the arrogance endemic to medical culture: the presumption that expertise confers a right to operate beyond ordinary inquiry. His corrective was not the erosion of professional judgment but its subjection to legitimate scrutiny. That standard, routine in peer‐reviewed research and outcomes reporting, has not been extended to the institutions and governments that govern the system. The CODE STEMI (ST‐elevation myocardial infarction) program described below makes the distinction operational: the time standard is published before the program runs, and performance against it is reported continuously, not after a death.

The distinction between proactive and reactive answerability is decisive. Reactive data, produced when a crisis demands it, serves the institution. Proactive mandatory reporting, published on a defined schedule against standards set in advance, serves the patient. Canada and the United States both operate predominantly on the reactive model: answerability triggered by catastrophe, absorbed into institutional response, and allowed to lapse when the media cycle moves on. The smoke detector and the fire alarm are both responses to danger; only one prevents the fire.

Accountability is the consequential response to attribution and answerability. It requires that those identified as responsible for decisions, who have been obligated to explain those decisions, face meaningful consequences when their exercise of fiduciary duty falls short. Without attribution and answerability as prerequisites, accountability becomes punitive theater: the scapegoating of visible individuals after catastrophic events while the structural conditions that produced those events remain undisturbed.

This inversion is not accidental. Accountability without attribution permits the appearance of governance rigor while insulating those who hold fiduciary responsibility from any obligation to explain themselves. Measurement proliferates; ownership does not. The fiduciary circuit fails not because accountability is absent but because it has been reached for without completing the prerequisites on which it depends.

The philosophical foundations of this framework run from Hippocrates through Maimonides and Spinoza to contemporary governance theory, each tradition converging on the same structural demand: the person (patient) before us is the primary reality, authority is held in trust rather than owned, and the only legitimate test of any governance arrangement is its effect on how patients actually live.^8^ Spinoza's concept of conatus, the drive of every institution to persist not merely in existence but *unchanged*, provides the mechanism: institutions resist structural governance reform not from malice but because such reform threatens their current configuration of authority and self‐interest. The fiduciary circuit does not demand virtue from those within it. It creates the structural conditions under which virtue becomes the rational choice.

The measurement architecture that would make the fiduciary circuit operational has a well‐established foundation. In these pages, Donabedian established in 1966 that quality evaluation requires attention to three domains: structure, process, and outcome, each necessary, none sufficient alone.^9^ The caution he expressed about the complexity of outcome measurement has been misinterpreted as a license to avoid outcome measurement altogether. The result is a quality infrastructure in which structural inputs are reported, process indicators are tracked, and outcomes, the ultimate validators of whether the enterprise is achieving its purpose, remain largely unmeasured, unpublished, and unaccountable. What Donabedian also understood is that quality evaluation is itself a governance enterprise: the decision about what to measure, how to measure it, and what consequences follow is not a technical question. It is a fiduciary question. Porter's value framework makes the same demand from a different direction: organize health care around patient medical conditions across the full cycle of care, measure outcomes achieved relative to resources consumed, and make results transparent and comparable.^10^ The argument is separated from Donabedian's by four decades and by discipline, but they are structurally identical. Both insist that the patient's condition across the full arc of their suffering and recovery is the only legitimate unit of moral and operational attention. Both are ignored, consistently, by systems that have arranged their measurement infrastructure around everything else.

## 3 Why the Circuit Fails: The Institutional Architecture of Resistance

Loss aversion explains much of the institutional resistance to reform. Kahneman and Tversky established that losses weigh roughly twice as heavily as equivalent gains in decision‐making,^11^ so institutions facing structural governance reform experience the threatened losses, to budget, authority, identity, and established practice, far more forcefully than the prospective gains, and resist accordingly. Thaler's sunk‐cost analysis is the close complement:^12^ decades of capital and professional identity invested in current arrangements are experienced not as inertia but as accumulated wisdom, and failure is reframed as the cost of admirable complexity.

Taleb's skin in the game formulation^13^ completes the diagnosis. Systems become compromised and fragile when those who make decisions are insulated from their consequences. The aviation parallel is structural: the pilot dies with the plane. That irreducible alignment between the operator's survival and the passenger's is the foundation of aviation's safety culture, not virtue, not training, not regulation, but physics. In health care governance, no equivalent alignment exists by default. The administrator who approves staffing cuts goes home. The minister who signs a bilateral agreement without binding outcome accountability moves to the next portfolio. The fiduciary circuit is the deliberate attempt to create, by design, the accountability that aviation gets from physics.

Vaughan's analysis of the Challenger disaster introduced the concept of normalization of deviance,^14^ the gradual institutional reclassification of risk signals as acceptable, not through negligence, but through the progressive development of interpretive frameworks that render signals manageable. In Canadian health care governance, this normalization operates at every level simultaneously. Wait times that would have been considered unacceptable a generation ago are now benchmarked as performance metrics, their absolute magnitude obscured by the normalizing framing of relative improvement. Hallway medicine, patients receiving care in corridors because no beds are available, has become a staffing metric rather than a moral emergency. In the United States, emergency department boarding, surgical backlog growth, and persistent rural access gaps have undergone the same reclassification: from crisis to management challenge, each threshold shift individually explicable, collectively representing a system that has drifted from its founding obligation.

Machiavelli observed the political geometry of this resistance five centuries before the behavioral economists named its mechanism: those who benefit from the existing order resolutely resist while those who might benefit from change remain uncertain and passive.^15^ The Mazankowski, Romanow, and Kirby reports in Canada, like the Institute of Medicine reports in the United States, each produced genuine analytical work, genuine short‐term improvement in selected metrics, and no durable change in the governance architecture that produced the need for the commissions in the first place. Each was analytically defensible. Each was absorbed by the same institutional architecture it was designed to challenge.

## 4 The Evidence: What the Circuit Looks Like When It Works, and When It Fails

Cardiac surgery provides the proof of concept for the fiduciary circuit at the program level. The development of the Society of Thoracic Surgeons outcomes databases, provincial cardiac registries, and risk‐adjusted mortality reporting created, across several decades, an environment in which attribution, answerability, and accountability became operationally embedded in everyday practice. Risk‐adjusted mortality rates declined. Complication profiles improved. Institutional variation in outcomes, once invisible, became visible and therefore tractable.^16^

The New York State experience with surgeon‐specific mortality disclosure in the early 1990s illustrates the importance of sequence.^17^ The initial effect was perverse: case selection shifted toward lower‐risk patients, protecting institutional metrics rather than serving the population. It took nearly a decade and the development of robust risk stratification for referral patterns to normalize and population‐level integrity to be restored. The limits of disclosure alone persisted even so: twenty years into public reporting, a study of New York cardiologists found the surgeon report cards little used in referral decisions, answerability that reached the public record without becoming consequential.^18^ The lesson was not that public reporting was wrong. It was that accountability imposed without the supporting framework of attribution and answerability generates perverse incentives before it generates improvement. The dependence matters. It always matters.

The same jurisdiction documents the failure mode in detail, as a counterfactual to CODE STEMI rather than an offset to it. The Canadian Institute for Health Information's dedicated Cardiac Care Quality Indicators project was discontinued in 2022, with no current plans to review or update the indicator methodology or the results. The governance architecture that would have ensured those findings produced structural consequences was never built: the instrument was retired and the oversight was not replaced. Locally, the cardiac outcome data infrastructure lapsed through successive withdrawals rather than by any single decision. The remote terminal link to the provincial population‐data center was removed; the contract of the statistical programmer who maintained it was not renewed; and the cardiac surgical database was discontinued, severing the final link between surgical outcomes and population data. The clinical‐administrative linkage between the Cardiac Sciences Program at St. Boniface Hospital and the Manitoba Centre for Health Policy, one of the world's most sophisticated health administrative databases, capable of linking surgical outcomes to population health trajectories across the full cycle of care, was dismantled not by decision but by institutional disappearance: no category in the government's research framework for clinical fiduciary infrastructure, and so no mechanism for protecting what could not be classified.^19^ The instrument that could answer Donabedian's^9^ question, not whether the operation was technically successful but whether the patient was genuinely better off and supported by both a clinical and administrative database, was lost to a category error. The capacity was not abolished by decision so much as allowed to lapse, which is precisely the failure mode this paper describes. What was built was not structurally protected, and so it was not retained.

The academic health center compounds this failure structurally, and the pattern is consistent across jurisdictions. Universities and medical schools are organized around disciplines, around what physicians do, rather than around what patients need across the full cycle of care. When departmental performance is measured by research output, trainee throughput, and disciplinary reputation, the institution is in structural conflict of interest with its fiduciary obligation to the patient. A recent analysis of academic medical center sustainability identified misaligned incentives as a core governance problem: academic medical centers “struggle with clinician engagement in part because incentives are misaligned,” with departmental productivity metrics systematically displacing patient outcome measurement as the primary performance standard.^20^ This is the fiduciary circuit failing at the institutional level, not through malice but through a governance architecture that rewards what disciplines produce rather than what patients need. The structural corrective is well‐established in principle and inconsistently applied in practice. The University of Calgary's Department of Cardiac Sciences formally integrates cardiology and cardiac surgery under unified academic governance, a single department, a single chair with protected authority, and a reporting structure that does not run through adjacent departments whose primary obligations lie elsewhere. The University of Leicester's Department of Cardiovascular Sciences provides an analogous model. These structures are not universal: most academic medical centers in Canada and the United States maintain separate departments whose governance does not protect integrated patient care as its primary obligation. The Cleveland Clinic, which is not a university, organizes its cardiovascular care into a single integrated institute rather than separate departments, and its standing as a world‐class academic program rests on that governance design rather than on academic affiliation. The question the fiduciary circuit asks of every academic health center is the same question it asks of every government and hospital: what does this institution exist to do, for whom, and what governance architecture ensures it does that rather than something else?

The same pattern operates at the system level. Canada's federal‐provincial health transfers are governed by the Canada Health Act against five principles but without binding outcome accountability for either party. The federal government holds the lever, the transfer, and its conditions, but does not deliver care; the provinces deliver care but are not answerable to the federal government for population outcomes. Responsibility for the outcome forms on neither side of that seam, and the incoherence lies not in the division of powers, which is internally consistent, but in the governance layer laid over it, which demands accountability for outcomes it has never made attributable. The bilateral agreements that followed, the 2003 Health Accord, the 2004 Ten‐Year Plan, the 2023 Working Together agreements, each announced targets, constituted working groups, and produced reports. None established the mechanism by which persistent nonperformance would produce a consequential response. The Parliamentary Budget Officer's assessment of the 2023 agreements found precisely what the preceding twenty years predicted: targets set, accountability absent, the transfer continuing regardless of whether the obligation it was meant to purchase was being met.^21^ The answerability is performative. The circuit remains open.

**Figure 2:**
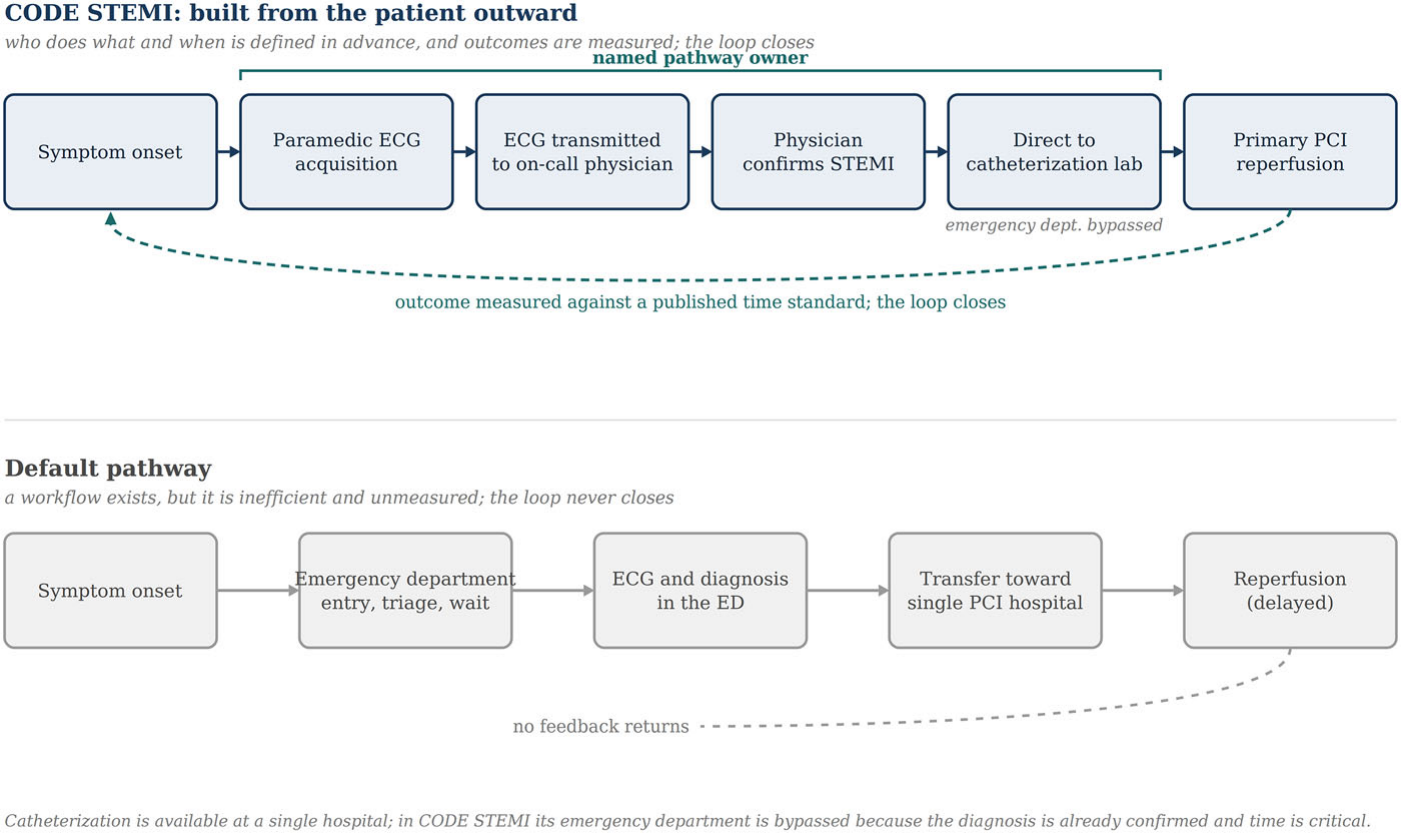
Two Pathways for Acute STEMI Care. Catheterization is available at a single hospital. In CODE STEMI a named owner carries the patient from onset through paramedic electrocardiogram (ECG) acquisition and transmission to on‐call physician confirmation; because the diagnosis is confirmed upstream and time is critical, the patient is taken directly to the catheterization laboratory and the emergency department is bypassed, with performance measured against a published time standard, closing the loop. In the default pathway the same care proceeds through an inefficient workflow that is not built from the patient outward: the diagnosis is made after the patient reaches the emergency department, which becomes the destination rather than a step, time is lost before definitive treatment, and performance is not measured against a standard, so the loop never closes. Figure rendered with the assistance of Claude Opus 4.8 (Anthropic), accessed June 2026, from the author's design and specification. All conceptual content, structure, and clinical accuracy were determined and verified by the author. [Colour figure can be viewed at wileyonlinelibrary.com]

Manitoba's CODE STEMI program demonstrates that the corrective is achievable at the program level, and it traces the circuit through all three functions. Developed collaboratively by cardiology, emergency medicine, and paramedic services, it defines a care pathway for patients experiencing a severe, time‐critical heart attack. Attribution is explicit: a named pathway owner carries the patient from paramedic electrocardiogram acquisition and transmission, through on‐call physician confirmation, to direct transport to the catheterization laboratory, bypassing the emergency department entirely. Answerability is built into the clock: a time standard published before the program was announced and measured continuously. Accountability follows: deviation from the standard is visible and correctable. A 2016 systematic review co‐authored by the author and colleagues from the Cardiac Sciences Program found prehospital electrocardiogram acquisition associated with a 32% relative risk reduction in mortality.^22^ The pathway worked because it was built from the patient outward, with the outcome defined before the pathway was designed and the result measured against that standard continuously (Figure 2). The question this paper asks of the broader governance architecture is why the same logic has not been applied at the system level.

The framework applies with equal force to waiting, the patient‐welfare problem Canada has grappled with for decades. The field has been moving from a queuing paradigm, in which the wait is dead time to be minimized, and the governing metric is median wait against a benchmark, toward navigation and optimization, in which the wait is preparation time to be used. The first is answerability without attribution: a number is reported, no one owns the patient's trajectory, and a long wait quietly reclassifies as within benchmark. The second restores the circuit. In an implemented and ever‐evolving local reform, a wait‐list office was reconstituted as an access and navigation function, and the wait was redefined around medical urgency and a clinical optimization timeline, metabolic stability, glycemic control, and management of anticoagulation among them, rather than around queue position alone. The navigation function also weighs the social and economic circumstances that bear on recovery, medication adherence, and post‐discharge support among them, since these influence the longer course of recovery alongside the procedure. This is consistent with consensus enhanced‐recovery recommendations in cardiac surgery^23^ and with evidence that timing, not mere speed, governs outcome. A recent analysis found the lowest mortality for coronary bypass performed three to seven days after catheterization for non‐ST‐elevation infarction,^24^ while local data showed that prolonged in‐hospital waits did not worsen outcomes for urgent bypass, and that patients operated on soonest required more blood transfusions and prolonged ventilation and spent longer in intensive care than those who waited several days, evidence that the interval functioned as optimization rather than delay.^25^ There is an optimal window, and both tails, excessive delay and premature operation, carry harm. In the framework's terms, the wait is attributed to a named office, answerable against a benchmarked clinical standard, and consequential when the standard is not met. The reform remains responsive to variable demand and infrastructure constraints and is offered as an instance of the circuit applied to queuing rather than as a finished solution.

## 5 The Corrective: Popperian Governance and the Architecture of Independence

The corrective is not another comprehensive reform agenda. Canada and the United States have both had those, and Machiavelli predicted their fate precisely. Nor is it a better‐designed commission: commissions authorized by incumbent institutions will continue to ask the questions those institutions are prepared to answer. The corrective is what Popper^26^ called piecemeal social engineering.

Two of Popper's ideas operate together here. Falsifiability requires that a governance arrangement be stated so that reality can refute it. Piecemeal engineering, governance by trial and error, then test the arrangement, and revise it incrementally in response to those refutations. A concrete instance carries both: this federal transfer will reduce a named wait time to a stated threshold by a stated date, and if it does not, a defined consequence follows. That is the standard already demanded of clinical practice, a claim framed so that reality can refute it, applied to governance, which ordinarily exempts itself from refutation. The unit of intervention is specific and testable, the commitment is to correction rather than continuity, and every governance arrangement is treated as a falsifiable hypothesis rather than an inherited fact.

Applied to the fiduciary circuit, this means three specific structural requirements. First, attribution: public governance documents must identify which institutions are responsible for which outcomes, not which institutions administer which processes. The assignment must be specific, testable against evidence, and revised when it proves inadequate. In federated systems, Canadian or American, this means that federal health transfers carry explicit attribution requirements: which level of government is responsible for which population outcomes, by when, measured how, with what consequence for persistent nonperformance.

Second, answerability: mandatory regular public reporting against standards set before the program is announced. Not reactive data produced when a crisis demands it, but proactive surveillance, the smoke detector rather than the fire alarm. The Australian Commission on Safety and Quality in Health Care provides a working comparator: an independent statutory authority with the power to set national standards, accredit health service organizations, and publish performance data publicly.^27^ Its mandate is insulated from the institutions it regulates. The Health Council of Canada, established after the Romanow Commission and defunded in 2014, demonstrated both the possibility and the political fragility of such a body: what distinguished its fate from the Australian model was not analytical capacity but structural independence, which the Health Council was never fully granted.^28^

The parallel in the United States is structural, and the single shared defect can be named: central transfers without binding outcome accountability. The federal government transfers funds to states through Medicaid and Medicare without binding outcome accountability for either party. States in turn contract with health systems and managed care organizations against process and utilization metrics that do not require outcome measurement across the full cycle of care. The Centers for Medicare and Medicaid Services has developed quality reporting infrastructure of genuine sophistication: Hospital Compare, the Merit‐based Incentive Payment System, and the Hospital Value‐Based Purchasing program. Each measures something real. None creates the structural conditions under which persistent nonperformance at the population level produces a consequential response from an independent body with the authority and mandate to enforce it. The Commission on Evidence‐Based Policymaking reported in 2017 that the federal government's capacity to evaluate whether its health programs are achieving their purposes is severely constrained by the fragmentation of data systems and the absence of binding outcome accountability in program design.^29^ The same description applies to Canada. The National Quality Forum, the Agency for Healthcare Research and Quality, and the Patient‐Centered Outcomes Research Institute each represent partial steps toward independent outcome evaluation. None has the statutory independence, the standard‐setting authority, or the enforcement mandate of the Australian Commission. The political will to grant that independence has not been present in either country, for the same structural reason: those who would grant it are the same institutions whose discretion it would constrain.

Third, accountability: consequences for persistent nonperformance that are proportionate, predictable, and enforced by bodies with genuine independence from the institutions they regulate. Not event‐driven responses to visible crisis but the continuous Popperian pressure of a governance system that treats underperformance as a correctable hypothesis rather than an inherited condition. Appropriate consequences mean that the response is scaled to the persistence and severity of nonperformance, not to its political visibility. Predictable consequences mean that institutions know in advance what failure to meet fiduciary obligations will produce. Independent enforcement means that the body empowered to assign consequences has no structural interest in the continued operation of the institutions it regulates, a condition that currently applies to almost none of the accountability mechanisms in place.

None of this requires new legislation in the first instance, though some will ultimately require it. What it requires first is the institutional architecture of independence: bodies empowered to attribute fiduciary responsibility, compel answerability, and enforce accountability, whose own mandate, funding, and membership are insulated from the institutions they regulate. To hold across the political cycle that creates it, such a body must have statutory permanence, its existence secured in legislation rather than in a renewable agreement that a later government can decline to renew. That independence is not an end in itself. It is the condition under which these bodies hold their duty where the fiduciary tradition places it: to patients and to populations, not to the institutions they oversee. Deming's observation applies equally to health governance as to manufacturing: every system is perfectly designed to get the results it gets.^30^ The results Canada and the United States are getting from their governance architectures are the results those architectures are designed to produce.

## 6 Conclusion: The Order of Dependence That Has Not Been Tried

Canada built a universal system on the premise that access should not be determined by ability to pay. The United States embedded the same premise in its major public programs, Medicare, Medicaid, and Veterans Affairs care, and extended it further, though partially and not without contest, through the Affordable Care Act. That premise remains admirable and, in a world of corrosive health inequity, increasingly important. The universal insurance model is not the problem. The problem is that the moral clarity of the founding premise has not been extended into the governance architecture that administers it. What was designed as a guarantee of access has drifted, institution by institution and agreement by agreement, into a guarantee of process: activity, reporting, the appearance of governance, while the outcomes that were the whole point quietly recede from view.

Donabedian established in 1966 that outcomes remain the ultimate validators of the effectiveness and quality of medical care.^9^ Roos and colleagues documented from Manitoba in 1996 that critical assessments of care typically focus on the clinical outcomes of individual treatments and the quality of care delivered by institutions, not on the health of populations.^19^ Nearly 60 years after the first observation and nearly 30 years after the second, the governance architecture that would make outcome measurement enforceable rather than informative has not been built. The fiduciary circuit, attribution, and answerability standing before accountability, is the structure that would build it.

The cardiac surgery experience demonstrates that completing the circuit produces measurable improvement. The New York State disclosure experience demonstrates that completing it in the wrong order induces perverse incentives before it produces improvement. The Manitoba governance record demonstrates that leaving it incomplete across three decades produces the same preventable failures documented repeatedly, accepted repeatedly, and not corrected at the structural level. The American commission record demonstrates the same pattern at a continental scale.

Governance design is not the only determinant of system performance; funding, workforce, and demography plainly matter. The narrower and defensible claim is that the specific, repeated failure documented here, the nonimplementation of recommendations that were correctly identified, is a governance failure of attribution and enforcement, and that it will recur until that architecture is built. The corrective does not require new analytical tools so much as the institutional architecture to act on the ones we already have: first, name who is responsible; second, require them to explain themselves against a standard set in advance; third, then and only then, hold them to account. That is what the fiduciary tradition has demanded since Hippocrates, and what every patient who places their life in the hands of a system built in their name has every right to expect.

*Attribution without answerability is paperwork*.

*Answerability without accountability is performative*.

*Accountability without attribution is punishment*.

*Canada and the United States have had all three*.

*Yet neither has honored the dependence among them*.

## Funding

No external funding was received for this work.

## Conflict of Interest Disclosures

The author declares no conflicts of interest.
